# Joint mobilization for frozen shoulder: A protocol for systematic review and meta-analysis

**DOI:** 10.1097/MD.0000000000029123

**Published:** 2022-04-08

**Authors:** Junjie Yao, Chang Liu, Tingting Pang, Jiahui Li, Siyuan Lei, Jiangchun Zhang, Yufeng Wang, Qiangqiang Shang

**Affiliations:** a Department of Acupuncture and Tuina, Changchun University of Chinese Medicine, Changchun, China; b Department of Tuina, The Affiliated Hospital to Changchun University of Chinese Medicine, China.

**Keywords:** frozen shoulder, joint mobilization, meta-analysis, rehabilitation, systematic review

## Abstract

**Background:**

The purpose of this study was to evaluate the effectiveness and safety of Joint mobilization in the treatment of frozen shoulder.

**Methods:**

To collect relevant literature, we will research following databases: Medicine, PubMed, Embase, Web of Science, Cochrane Library, China National Knowledge Infrastructure, Wan-Fang Database, Chongqing VIP Chinese Science and Technology Periodicaols Database, and China Biomedical Database; the time is from its creation to May 2021, and the language is limited to Chinese and English. In addition, we will retrieve other literature resources, including the Chinese Clinical Trial Register and conference articles. Two reviewers will independently complete the literature screen and data extraction and quality assessment of the included studies will be independently completed by 2 other researchers. The primary outcomes included joint ROM and Japanese Orthopedic Association score. Visual Analogue Scale score, Activities of Daily Living score and adverse reactions as secondary outcomes were assessed. RevMan V.5.4.1 software will be used for meta-analysis, and the Grading of Recommendations Assessment, Development and Evaluation (GRADE) will be used to assess the quality of evidence.

**Results:**

This systematic review will provide a high-quality synthesis to evaluate the efficacy and safety of joint mobilization in the treatment of frozen shoulder, providing a reference for the safe and effective treatment of frozen shoulder.

**Conclusion:**

This study provides evidence that joint mobilization in the treatment of frozen shoulder is effective.

**Ethics and dissemination:**

The protocol of the systematic review does not require ethical approval because it does not involve humans. This article will be published in peer-reviewed journals and presented at relevant conferences.

**Systematic review registration:**

INPLASY202210075

## 1 Introduction

Frozen shoulder is a clinical syndrome characterized by shoulder pain and mobility dysfunction due to soft tissue lesions around the shoulder joint.^[1]^ The literature suggests that the global prevalence of frozen shoulder is 2% to 5%.^[2]^ Most people who develop frozen shoulder are between 40 and 60 years of age^[3]^ and with the exception of secondary traumatic frozen shoulder,^[4]^ it is uncommon in patients over 70 years of age. The incidence in women is 1.6 compared to men.^[5]^ A variety of frozen syndromes exist clinically, with frozen shoulder being the most common “frozen” syndrome.^[6–8]^ The exact etiology of frozen shoulder is unknown, and the disease is presently thought to be the result of a combination of synovial inflammation and joint capsule fibrosis. The clinical symptoms of the disease are mostly pain in the shoulder, with severe pain at night or even insomnia, and severe limitation of shoulder movement, especially abduction and external rotation, often in a state of “freezing, condensation, and knotting,” hence the name frozen shoulder. Present studies have found that a large number of patients with frozen shoulder do not spontaneously subside.^[9]^

The common nonsurgical treatments include medication, physical therapy, exercise, manipulation under anesthesia, steroid injections, or nerve blocks, which can temporarily relieve symptoms, but have inaccurate results, are prone to recurrence, have drug side effects, and have long treatment intervals and poor patient compliance.^[10,11]^ The Mulligan joint release procedure is a new manipulative technique created by Brain R Mulligan for joint Mulligan joint release is a new manipulative technique created by Brain R Mulligan for joint dysfunction, which can restore normal joint gliding and improve symptoms within a short treatment period.^[12]^ Arthrodesis is highly valued by patients for its efficacy, ease of use, and excellent prognosis. Although many clinical studies have reported good efficacy of arthrodesis in the treatment of frozen shoulder, there is not sufficient clinical evidence. Therefore, this systematic review aims to evaluate the effectiveness and safety of arthrodesis for frozen shoulder and to provide a better basis for clinical decision making.

## 2 Methods and analysis

The study was conducted following the guidelines of the Preferred Reporting Items for Systematic Review and Meta-analysis Protocol (PRISMA-P).^[13]^ This study protocols have been funded through a protocol registry. This protocol of the systematic review has been registered on the INPLASY website. Registration: INPLASY202210075.

### 2.1 Inclusion criteria

#### 2.1.1 Types of participants

The inclusion criteria were patients aged >18 years with shoulder pain and restriction in range of motion. A symptom duration >3months was required, with no radio graphic findings on anteroposterior shoulder plain radiographs except for osteoporosis. No medical treatment, other than analgesics, was prescribed within the past 3 months. Patients were excluded from the study if they were pregnant, if they had had surgical intervention on the affected shoulder, if there was extensive scar around the shoulder, rotator cuff calcification, joint infection, lack of stability, rheumatoid arthritis or full thickness tear of shoulder rotator cuff, cervical radiculopathy or damage to the spinal cord, or history of cortisone injection in the affected area in the previous 6 weeks, or if they had other contraindications to shock wave treatment, including artificial pacemaker, use of anti-blood clotting medications, known bleeding disorder, known malignancy in the area intended for treatment, or epilepsy.

#### 2.1.2 Types of interventions

The interventions in the experimental group included only joint mobilization. It mainly included different levels and types of joint mobilization. The interventions of the control group should only be rehabilitation therapy. If there are other adjuvant therapies, the 2 groups should be consistent.

#### 2.1.3 Types of studies

Inclusion: We will include only randomized controlled clinical trials (RCTs) of joint mobilization for frozen shoulder.

Exclusion: We will exclude any other literature including non-randomized clinical controlled trials, retrospective research literature, conference abstracts, case reports, repeated published literature, and literature of information without data.

#### 2.1.4 Types of outcomes

##### 2.1.4.1 Main outcomes

We will include the joint range of motion and the Japanese Orthopedic Association score as the main outcomes. The range of motion will be used to assess the patient's shoulder mobility. The Japanese Orthopedic Association will assess the patient's shoulder motor function, with higher scores indicating better functional status.

##### 2.1.4.2 Secondary outcomes

1. Activities of Daily Living score is used to assess the ability to perform daily living in stroke patients.
2. Visual Analogue Scale score is used to assess the degree of pain in patients with frozen shoulder.
3. Adverse effects.

### 2.2 Data sources and search methods

#### 2.2.1 Electronic searches

We will collect relevant articles by searching the following databases: PubMed, Web of Science, Medicine, EMBASE, Cochrane Library, China National Knowledge Infrastructure, China Biomedical Literature Database, China Science Journal Database, and Wan-Fang Database. All databases will be searched from creating to Jan.16, 2022, by the following words: Frozen Shoulder∗, Shoulder Joint, Shoulder Pain, Adhesive Capsulitis of the Shoulder, Shoulder Adhesive Capsulitis, Rotator Cuff Injuries, Mulligan's mobilization∗, Joint mobilization, Joint Release, Physical Therapy Modalities, Mobilization with Movement, Rehabilitation, Habilitation, RCT, among others. The research strategy for PubMed is presented in Table 1.

**Table 1:** Search strategy used in PubMed.

No	Search Items
#1	Frozen Shoulder^∗^ (All Fields)
#2	Shoulder Joint (All Fields)
#3	Shoulder, Frozen (All Fields)
#4	Shoulder Pain (All Fields)
#5	Adhesive Capsulitis of the Shoulder (All Fields)
#6	Shoulder Adhesive Capsulitis (All Fields)
#7	Rotator Cuff Injuries (All Fields)
#8	Adhesive Capsuliti^∗^ (All Fields)
#9	#1 OR #2-#8
#10	Mulligan's mobilization^∗^ (All Fields)
#11	Joint mobilization (All Fields)
#12	Joint Release (All Fields)
#13	Physical Therapy Modalities (All Fields)
#14	Mobilization with Movement (All Fields)
#15	Rehabilitation (All Fields)
#16	Habilitation (All Fields)
#17	#10 OR #11-#16
#18	Randomized controlled trial (All Fields)
#19	Controlled clinical trial (All Fields)
#20	Randomized (All Fields)
#21	Randomly (All Fields)
#22	#18 OR #19-#21
#23	#9 AND #17 AND #22

#### 2.2.2 Searching for other resources

We will search the reference list of the included studies and existing systematic reviews related to our topic. We will also search for other literature resources, including the Chinese Clinical Trial Register, conference articles, and other related gay literature to make our search as complete as possible.

### 2.3 Data collection and export

**Figure 1:**
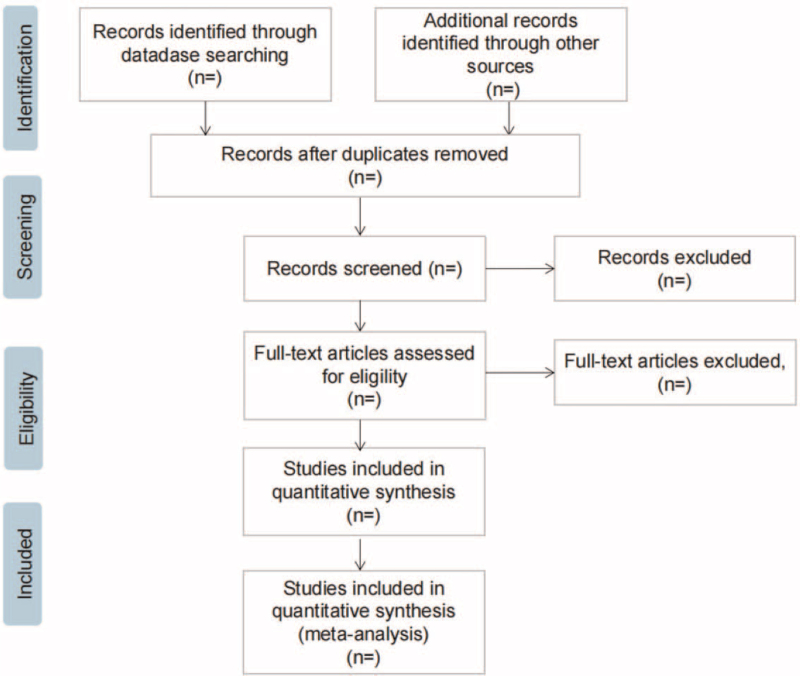
Flow diagram of study selection process.

Two researchers independently screened the literature according to the eligibility criteria. First, they eliminated duplicate articles using EndNote V.x 9.0, and excluded articles that did not meet the inclusion criteria by reading the title and subject. Second, they will perform a screen again of the remaining articles by reading the full text according to the inclusion and exclusion criteria and determine whether it is available for the systematic review. We will also record the excluded papers and explain the reasons for this; the specific screening process is shown in Figure 1. If there is disagreement during, the third researcher will be invited to make a decision.

### 2.4 Data extraction and analysis

Data extraction will be performed by two reviewers independently, and the results will be cross-matched. When the differences and opinions are inconsistent, they should be settled through discussion. If the differences encountered cannot be resolved through discussion, a third researcher will be invited to resolve them. We will make an Excel to extract data which includes the first author, country, year of publication, patient characteristics, number of participants, interventions, outcomes, results, main conclusions, conflicts of interest, ethical approval, and other information. If necessary, we will contact the corresponding author by e-mail to obtain more accurate data.

### 2.5 Assessment of risk of bias in the included studies

Two researchers will independently evaluate the bias risk, including studies using the assessment tool of risk bias in the Cochrane Handbook V.5.1.0..^[14]^ The contents included random sequence generation, allocation sequence concealment, blinding of participants and personnel, outcome assessors, incomplete outcome data, selective outcome reporting, and other sources of bias. The assessment results will be rated as low-risk, high-risk, and uncertain risk. In the process, if there is disagreement, a third reviewer will be invited to make a decision.

### 2.6 Assessment of heterogeneity

The heterogeneity test will be carried out among all studies included using the *I*^2^ statistic. When *I*^2^ was <50%, there was no significant heterogeneity. Otherwise, if the result of *I*^2^ is >50%, we believe that there is obvious heterogeneity and subgroup analysis and sensitivity analysis will be conducted to investigate the sources of heterogeneity.

### 2.7 Assessment of reporting biases

We will analyze the quality of publication bias using Rev Man 5.4.1 software in inverted funnel plots and performing Egger test when there were >10 trials included in the meta-analysis.

### 2.8 Data synthesis

The meta-analysis of data from included outcomes will be performed using the Rev Man V.5.4.1, and we will choose a randomized or fixed effect model for data statistics according to the results of the heterogeneity test. The enumeration data were expressed as relative risk, and the weight mean difference was used as the measurement data; each effect amount was expressed in 95% confidence interval. The specific methods were as follows: If the heterogeneity was low (*I*^2^ < 50%, the fixed-effects model was used for data synthesis. If there is high heterogeneity (*I*^2^ > 50%), the random-effects model will be used for data synthesis after excluding possible heterogeneity sources. The investigation methods included subgroup and sensitivity analyses. If data cannot be synthesized, we provide a descriptive analysis to solve this problem.

### 2.9 Subgroup analysis

If there was high heterogeneity (*I*^2^ > 50%) among the included studies, we conducted a subgroup analysis to analyze the sources of heterogeneity according to the following factors: age, sex, race, courses, sample sizes, different levels and types of joint mobilization, and other possible factors affecting the results.

### 2.10 Sensitivity analysis

To test the stability and reliability of the results of this study, we conducted a sensitivity analysis according to the following points: method quality, sample size, and missing data. After that, we will perform a data analysis again and compare the results. If there was no directional change after the sensitivity analysis, the results were stable.

### 2.11 Grading the quality of evidence

We will use the Grading of Recommendations Assessment, Development, and Evaluation to access confidence in cumulative evidence.^[14]^ The risk of publication, heterogeneity, indirectness, imprecision, and publication bias were assessed, and the results were divided into 4 levels: high, moderate, low, and very low.

### 2.12 Ethics and dissemination

Ethical approval will not be required, as no primary information of individual patients was collected. We will publish this article in a peer-reviewed journal.

## 3 Discussion

It is presently believed that the etiology of frozen shoulder may be related to the structural and physiological characteristics of the shoulder joint, acute injury (sprain and contusion, dislocation, among others), inflammatory stimulation, strain degeneration and endocrine disorders, which in turn cause joint pain, resulting in limited mobility and function and seriously affecting the quality of life of patients.^[15,16]^ Some studies have shown that shoulder joint dysfunction is closely related to rotational stiffness and the degree of pain affects joint mobility and function.^[17]^ It has been shown that Mulligan joint release with low frequency rolling and sliding can increase pain-free range of motion; however, it can reduce muscle tension in the relevant area, which in turn reduces pain, improves treatment outcome, and restores patients’ quality of life.^[18]^ However, due to the lack of evidence, this conclusion still needs to be supported by valid evidence. In this study, a systematic review and meta-analysis of data from relevant randomized controlled trials will be conducted to verify its effectiveness and safety and provide evidence-based medical evidence for the clinical treatment of this disease.

## Author contributions

Qiangqiang Shang and Junjie Yao contributed to the conception of this study. Junjie Yao drafted and revised the manuscript. The search strategy was developed by all the authors and will be performed by Chang Liu and Jiahui Li, Jiangchun Zhang and Tingting Pang will independently screen the potential studies and extract data from the included studies. Assess the risk of bias and complete Yufeng Wang. Junjie Yao will complete data synthesis. Qiangqiang Shang arbitrate disagreements. All authors approved the publication of the protocol.

**Data curation:** Chang Liu.

**Formal analysis:** Tingting Pang, Jiangchun Zhang, Qiangqiang Shang.

**Funding acquisition:** Qiangqiang Shang.

**Investigation:** Yufeng Wang.

**Methodology:** Jiahui Li, Siyuan Lei.

**Validation:** Junjie Yao, Qiangqiang Shang.

**Writing – original draft:** Junjie Yao.

**Writing – review & editing:** Qiangqiang Shang.
